# The subsurface Pt-promoted TiO_2−x_ overlayer enhances succinonitrile production in the photocatalytic C–C coupling of acetonitrile

**DOI:** 10.1093/nsr/nwaf588

**Published:** 2025-12-27

**Authors:** Xian Zhou, Houhong Song, Zhitong Chen, Shufang Zhao, Junting Wang, Yiou Wang, Xiaofeng Gao, Lili Lin, Siyu Yao

**Affiliations:** Key Laboratory of Biomass Chemical Engineering of Ministry of Education, College of Chemical and Biological Engineering, Zhejiang University, Hangzhou 310058, China; Key Laboratory of Biomass Chemical Engineering of Ministry of Education, College of Chemical and Biological Engineering, Zhejiang University, Hangzhou 310058, China; Key Laboratory of Biomass Chemical Engineering of Ministry of Education, College of Chemical and Biological Engineering, Zhejiang University, Hangzhou 310058, China; Key Laboratory of Biomass Chemical Engineering of Ministry of Education, College of Chemical and Biological Engineering, Zhejiang University, Hangzhou 310058, China; School of Interdisciplinary Science, Beijing Institute of Technology, Beijing 102488, China; School of Interdisciplinary Science, Beijing Institute of Technology, Beijing 102488, China; Key Laboratory of Biomass Chemical Engineering of Ministry of Education, College of Chemical and Biological Engineering, Zhejiang University, Hangzhou 310058, China; Institute of Zhejiang University-Quzhou, Quzhou 324000, China; Institute of Industrial Catalysis, State Key Laboratory of Green Chemistry Synthesis Technology, College of Chemical Engineering, Zhejiang University of Technology, Hangzhou 310014, China; Key Laboratory of Biomass Chemical Engineering of Ministry of Education, College of Chemical and Biological Engineering, Zhejiang University, Hangzhou 310058, China; Institute of Zhejiang University-Quzhou, Quzhou 324000, China

**Keywords:** photocatalysis, C–C coupling, radical reaction, reverse catalyst

## Abstract

Photocatalytic coupling of monofunctional molecules offers an atom-efficient route for the synthesis of value-added bifunctional organic compounds, yet its efficiency is significantly limited by the reverse reaction of radicals. Our density functional theory (DFT) calculations have indicated that a unique structure of partially exposed Pt encapsulated by a titanium oxide (TiO_2_) overlayer could intrinsically facilitate the desorption and suppress re-adsorption of reactive radicals, hence impeding the reverse reaction in the photocatalytic acetonitrile coupling reaction. A TiO_2−x_/Pt inverse heterostructure has then been developed via strong metal–support interaction (SMSI) with tunable TiO_2−x_ coverage. Among the catalysts, an optimal partially encapsulated TiO_2−x_/Pt catalyst achieves a marked formation rate of succinonitrile of 8.41 mmol·g_cat_^−1^· h^−1^ from acetonitrile, reaching a 67.3% radical-to-product efficiency and a 5.6% apparent quantum yield, representing 3-fold enhancements over a conventional Pt-supported TiO_2_ catalyst, over 1.9-fold higher than bare Pt/TiO_2_ or fully encapsulated counterparts, respectively. Kinetic investigations demonstrate that the suppression of radical–proton recombination plays a more dominant role in the overall coupling performance compared to the radical initiation. This work underscores the critical role of tailored catalysts by coating with oxide domains to mitigate reverse reactions and establishes an effective strategy for advancing the efficiency in photocatalytic coupling.

## Teaser

A universal strategy of tailoring oxide overlayers on metals offers a decisive solution to suppress reverse reactions, unlocking high efficiency in photocatalytic radical coupling.

## INTRODUCTION

The coupling of small monofunctional molecules (e.g. CH_3_X) to form value-added bifunctional compounds (e.g. XCH_2_CH_2_X) represents an important chemical engineering process for the production of industrial raw materials, such as ethylene glycol, succinonitrile (SN) and succinic acid. Unlike the complicated multi-step conventional organic synthetic approaches, photocatalytic coupling reactions over semiconductor-based heterogeneous catalysts have emerged as an attractive alternative [1–4]. Upon light excitation, the photoholes solely, or the hydroxyl radicals generated by photoholes, can preferably cleave the C_α_–H bond in monofunctional molecules (e.g. CH_3_X), forming reactive radical intermediates (e.g. CH_3_X→·CH_2_X) that subsequently realize the C–C coupling. As such, ethylene glycol [5], SN [6], succinic acid [7], etc. have been successfully produced in a highly atom- and step-economic manner from splicing abundant inexpensive methanol, acetonitrile and acetic acid precursors, respectively.

Despite the success achieved in the synthetic innovations, the net accumulation rates of the C–C coupling products remain limited by both the moderate generation rates of reactive free radicals from small molecules, and the reverse reactions where the initiated radicals are quenched by protons and photoelectrons instead of forming target products [8]. Optimization of the activity, selectivity and quantum efficiency of current photocatalytic systems is vital to advance photocatalytic coupling performance toward practical implementation [9,10].

In previous studies, substantial progress has been made in enhancing selective radical initiation over sulfide and TiO_2_ photocatalysts for the dimerization of alcohols, nitriles and lignin derivatives [5,6,11,12]. In comparison, suppression of the adverse surface reverse reactions to avoid the consumption of excited charge carriers and the depletion of the reactive free radicals has so far been overlooked to some extent and remains a formidable challenge. Recently, Gao *et al.* reported that modifying the hydrogen-bond network via water addition in ethanol promotes the desorption of α-hydroxyethyl radicals (CH_3_·CHOH) and thereby reduces the possibility of reverse reactions on Au/CdS, improving the productivity and selectivity of 2,3-butanediol by 2.4 and 1.5 times, respectively [13]. This pioneering work highlighted the importance of promoting the desorption of free radicals from the surface of a heterogeneous photocatalyst to suppress the reverse reaction. However, such a method requires changing the composition of the reaction solvent by adding water, restricting its applications within aqueous systems [6,7,14]. Therefore, a more universal strategy to develop a catalyst that intrinsically mitigates the surface reverse reaction is highly desired.

In the photocatalytic overall water splitting (OWS) reaction, coating the noble metal cocatalyst with inert oxide overlayers, such as CrO_x_ [15], SiO_2_ [16] and Al_2_O_3_ [17] and so on [18–21] is an efficient approach to suppress the undesirable reverse reaction of H_2_ oxidation on the metal surface. Recently, using the atomic layer deposition (ALD) technique to precisely control the coverage of the inert oxide layer of Al_2_O_3_ on the photocatalyst, Li *et al.* discovered that the unfavorable reverse reactions, including 2H_2_ + O_2_ → 2H_2_O and O_2_ + 4H^+^ + 4e^−^ → 2H_2_O, were successfully suppressed [17]. The Al_2_O_3_-decorated Rh/GaN–ZnO catalyst also achieved an apparent quantum yield of 7.1% for the OWS reaction at 420 nm, 23 times higher than that of the uncoated catalyst. As such, modifying the metal active sites in the photocatalyst with appropriate oxide overlayers could also inhibit the reverse reactions of free radicals in photocatalytic coupling reactions, hence dramatically enhancing the light-to-chemical efficiency.

In this work, a density functional theory (DFT) calculation was first used to predict that the interface of a TiO_2−x_/Pt inverse heterostructure reduces the adsorption energy (*E*_ads_) of ·CH_2_CN radicals, which facilitates their desorption into bulk solution and inhibits their re-adsorption simultaneously. Inspired by such knowledge, we demonstrate fabrication of a TiO_2−x_/Pt inverse heterostructure through a strong metal–support interaction (SMSI)-driven encapsulation process and show that the TiO_2−x_/Pt catalyst effectively suppressed the reverse reaction between ·CH_2_CN radicals and protons. An optimized partially encapsulated TiO_2−x_/Pt inverse interface exhibits exceptional performance, with the SN formation rate reaching 8.41 mmol · g_cat_^−1^·h^−1^, 1.9-fold higher than both bare and fully encapsulated counterparts of Pt/TiO_2_. With the efficient suppression of the reverse reaction, the partially encapsulated Pt/TiO_2_ catalyst (TiO_2−x_/Pt inverse structure) achieves 67.3% radical-to-product efficiency and 5.6% apparent quantum yield at 365 nm, a 3-fold improvements over counterparts. The quantitative analysis reveals that suppressing proton/electron-mediated ·CH_2_CN radical recombination is more crucial for overall productivity than promoting radical initiation in the acetonitrile coupling reaction. This work paves a new way to improve the reaction efficiency of photocatalytic coupling reactions from small organic molecules to value-added bifunctional compounds.

## RESULTS AND DISCUSSION

### Theoretical prediction of the adsorption energy of active free radicals on the surface of the Pt/TiO_2_ model surfaces

**Figure 1.**
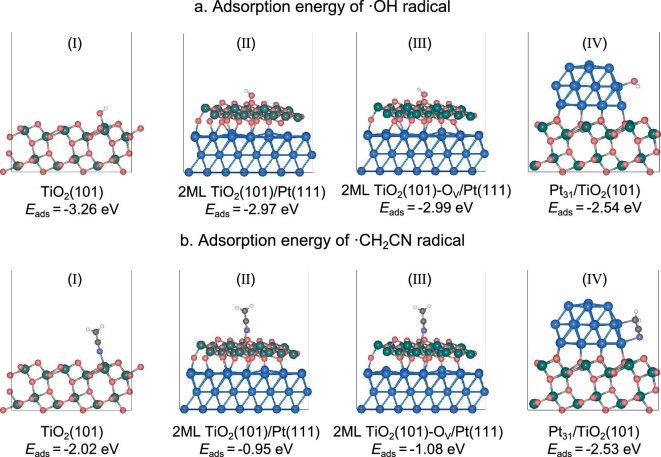
Theoretical prediction of the *E*_ads_ of reactive radicals on the surface of Pt-TiO_2_ systems. The adsorption configuration and corresponding *E*_ads_ of (a) ·OH and (b) ·CH_2_CN on TiO_2_(101) (I), 2ML TiO_2_(101)/Pt(111) (II), 2ML TiO_2_(101)-O_v_/Pt(111) (III) and Pt_31_/TiO_2_(101) (IV) model structures. In the models, the C, H, O, N, Ti and Pt atoms are marked using gray, white, red, purple, dark green and blue colors.

Reducing the binding energy of reactive free radicals on the photocatalyst surface can promote their desorption after generation and simultaneously mitigate their re-adsorption possibility. Thus the adsorption/desorption behavior of hydroxyl radicals (·OH) and cyanomethyl radicals (·CH_2_CN), the main free radicals in the photocatalytic dehydrogenative acetonitrile coupling reaction, were first investigated on the Pt/TiO_2_ models using a DFT calculation (Fig. 1) [22,23]. The anatase-TiO_2_(101) surface models were constructed to represent the TiO_2_ semiconductor (Ⅰ). Pt_31_/TiO_2_(101) (Ⅳ) and two-monolayer (2ML) TiO_2_(101)/Pt(111) (Ⅱ) are used as the model of the exposed Pt/TiO_2_ interface and the TiO_2−x_/Pt inverse interface frequently appearing on the Pt/TiO_2_ catalysts, respectively. Based on the mechanism of photocatalytic dehydrogenative coupling of acetonitrile, ·OH is generated via the oxidation of water by the photoholes (H_2_O + h^+^ → ·OH + H^+^) [6], while the reactive ·CH_2_CN is formed through the H-abstraction of the C_α_–H bond by ·OH (CH_3_CN + ·OH → ·CH_2_CN + H_2_O). Considering the spatial distribution of electrons (migrating to the Pt co-catalyst) and photoexcited holes (remaining on the TiO_2_ surface), it is anticipated that the oxidation product ·OH radicals tend to form on TiO_2_. In contrast, the reverse reaction (H^+^ + e^−^ + ·CH_2_CN → CH_3_CN; H^+^ + e^−^ + ·OH → H_2_O), which requires the reduction of radicals, tends to occur at the Pt and interfacial site. Therefore, the desorption of generated reactive radicals from the hole of the semiconductor are compared between TiO_2_(101) (conventional Pt/TiO_2_) and 2ML TiO_2_(101)/Pt(111) surfaces (inverse interface) (Fig. 1). The calculation results show that on the surface of TiO_2_ and all Pt/TiO_2_ heterostructures, ·OH generally exhibits a stronger adsorption behavior than ·CH_2_CN, suggesting that the ·CH_2_CN can desorb more easily than ·OH. Thus, the *E*_ads_ (·CH_2_CN) is mainly used to quantitatively distinguish the suitable structure of the photocatalysts to promote the desorption of the ·CH_2_CN.

According to the theoretical calculations, the introduction of Pt beneath the TiO_2_(101) surface [forming 2ML TiO_2_(101)/Pt(111)] reduces the *E*_ads_ of ·OH from −3.26 to −2.97 eV and more strikingly decreases the *E*_ads_ of ·CH_2_CN from −2.02 to only −0.95 eV. Therefore, the inverse interface with Pt partially buried in TiO_2_ can accelerate the previously challenging desorption of radicals, particularly for the ·CH_2_CN radical, while the formation of oxygen vacancies at the TiO_2_/Pt interface only slightly influences the adsorption of radicals (Ⅲ). The adsorption of the reactive radicals on the Pt nanoparticles (NPs)/TiO_2_(101) is also calculated to determine the re-adsorption possibilities. The calculation results show that the *E*_ads_ of ·OH and ·CH_2_CN on Pt NPs is −2.54 and −2.53 eV, respectively. The relatively smaller *E*_ads_ of ·OH on Pt NPs demonstrates that ·OH radicals tend to re-adsorb back to the surface of TiO_2_ instead of Pt NPs. On the contrary, the ·CH_2_CN radical has the highest *E*_ads_ on the surface of Pt, which will induce a pronounced reverse reaction on the exposed Pt surface. Therefore, reducing the exposed surface area of Pt with overlayers of TiO_2_ will also suppress the re-adsorption and further inhibit the reverse reaction of reactive ·CH_2_CN with protons and excited electrons on the surface of the Pt co-catalyst. The projected density of states (pDOS) analysis reveals a crucial electronic interaction at the TiO_2_/Pt inverse interface (Fig. S1). In model (III), a distinct hybridization between the Pt 5*d* orbitals and the Ti 3*d* orbitals of the overlayer is observed, accompanied by an upward shift of the Pt d-band center relative to the fully exposed Pt NPs in model (IV). This electronic structure modification weakens the adsorption of radical intermediates, consistent with our DFT-calculated lower *E*_ads_.

### Synthesis and characterization of photocatalysts

Inspired by theoretical insights, we synthesized Pt/anatase-TiO_2_ photocatalysts featuring tunable TiO_2_ overlayer coverage on Pt co-catalysts through a classic process of SMSI-driven encapsulation [21,24,25]. The Pt/TiO_2_ precursor was prepared using a light-assisted deposition-precipitation method with chloroplatinic acid (H_2_PtCl_6_) and anatase TiO_2_ [20], yielding a platinum loading of approximately 1.5%, as determined by inductively coupled plasma atomic emission spectroscopy (ICP-AES) (Table S1). Following separation and calcination, the Pt/TiO_2_ precursor was reduced under hydrogen flow at temperatures ranging from 200°C to 600°C to activate the SMSI-driven encapsulation, enabling precise modulation of the coverage of TiO_2_ overlayer on the Pt NPs. The obtained photocatalysts were denoted as Pt/TiO_2_-*n*H, where *n* represents the hydrogen treatment temperature (°C).

**Figure 2.**
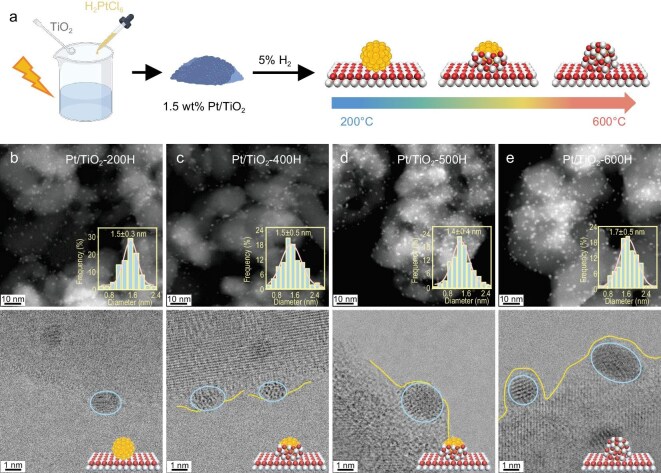
(a) Schematic presentation for the preparation of Pt/TiO_2_ photocatalysts and (b–e) TEM images. HAADF-STEM images and high-magnification STEM-ADF images of (b) Pt/TiO_2_-200H, (c) Pt/TiO_2_-400H, (d) Pt/TiO_2_-500H and (e) Pt/TiO_2_-600H (size distribution of Pt NPs and models of different catalysts are shown in the insets).

High-angle annular dark-field scanning transmission electron microscopy (HAADF-STEM) images and Pt NP size distribution analyses of the Pt/TiO_2_ catalysts (Fig. 2b–e) reveal that Pt NPs loaded in all samples have a similar average size of approximately 1.5 nm (Table S1). At the reduction temperature of 200°C, the Pt NPs remain fully exposed, as this temperature is insufficient to trigger the migration of TiO_2−x_ species (Fig. 2b and Fig. S2) [26,27]. As the reduction temperature increases, TiO_2−x_ species begin to migrate, forming overlayers that progressively cover the surface of Pt NPs. This results in an expanding TiO_2−x_/Pt inverse interface, correlated with rising TiO_2−x_ coverage (Fig. 2d, g and Fig. S3). Upon reduction at 600°C, the Pt NPs become completely encapsulated by amorphous TiO_2−x_ overlayers (Fig. 2e and Fig. S4).

**Figure 3.**
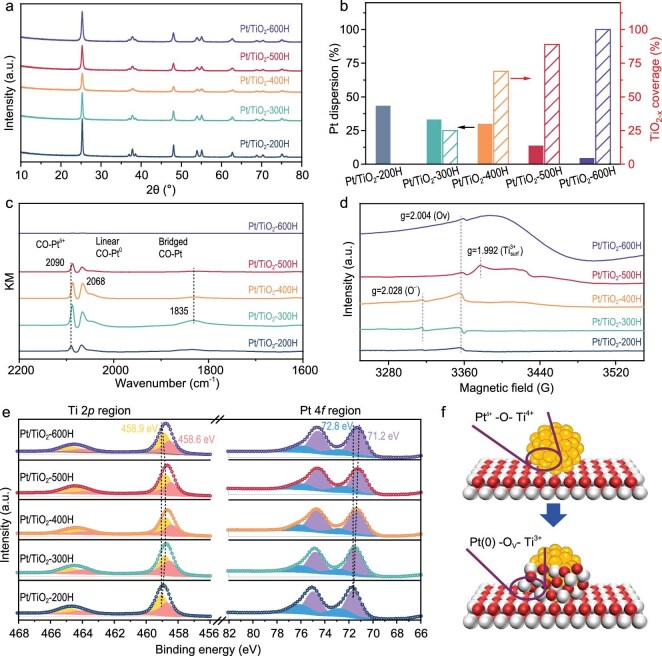
Structure characterization of Pt/TiO_2_ photocatalysts. (a) XRD pattern; (b) Pt NP dispersion and TiO_2−x_ interface coverage; (c) *in situ* CO-adsorption FTIR spectra; (d) EPR spectra; (e) Ti 2*p* and Pt 4*f* XPS spectra of Pt/TiO_2_-200H, -400H, -500H and -600H. (f) Schematic diagram of defects generated at the TiO_2−x_/Pt interface from 200°C to 500°C.

The structural evolution of Pt/TiO_2_ catalysts during hydrogen reduction was then systematically investigated via thorough characterizations. Powder X-ray diffraction (XRD) patterns of the Pt/TiO_2_-*n*H series exclude the agglomeration of Pt NPs during the H_2_ reduction, and the anatase phase of the TiO_2_ support remains intact (Fig. 3a). CO chemisorption measurements reveal a decline in the dispersion of Pt NPs from 43.5% (Pt/TiO_2_-200H) to 5.6% (Pt/TiO_2_-600H) with increasing reduction temperature, suggesting that the inverse interface coverage on Pt nanoparticle surfaces increased from 0% to 90% (Fig. 3b). CO probe diffuse reflectance infrared Fourier transform spectroscopy (DRIFTS) identified three distinct CO adsorption modes, namely the linearly coordinated CO on partially oxidized Pt^δ⁺^ at the metal-oxide interface (2090 cm^−1^), linear CO on metallic Pt(0) (2068 cm⁻^1^) and bridged CO on Pt surfaces (broad peak at ∼1835 cm^−1^) (Fig. 3c) [28–30]. The formation of TiO_2−x_ overlayers suppressed all three adsorption peaks. Notably, the CO-Pt^δ⁺^ peak redshifted by 3–4 cm^−1^ after reduction, indicating enhanced electron density at the interfacial Pt sites due to the H_2_ reduction [21].

Electron paramagnetic resonance (EPR) spectroscopy, sensitive to defects in heterogeneous catalysts, further elucidated interfacial restructuring in the Pt/TiO_2_ catalysts (Fig. 3d). All catalysts exhibited a signal at *g* = 2.004, attributed to oxygen vacancies (O_V_) with single electrons. As reduction temperatures rose, a new signal emerged at *g* = 2.028, intensifying alongside O_V_s, consistent with interfacial oxygen removal. Reduction at 500°C introduced an additional peak at *g* = 1.992, assigned to surface Ti^3+^ species, which corresponds to the reduction of interfacial Ti cations [31]. At 600°C, abundant bulk defects formed inside the support, acting as charge-carrier traps that diminished the redox capability of the TiO_2_ semiconductor [32]. X-ray photoelectron spectroscopy (XPS) of Ti 2*p*, Pt 4*f* and O 1*s* regions provided electronic insights into the TiO_2−x_/Pt inverse interface (Fig. 3e and Fig. S5). In Ti 2*p* spectra, Ti^4+^ (2*p*_3/2_: 459.1 eV) and Ti^3+^ (2*p*_3/2_: 458.7 eV) peaks were resolved for Pt/TiO_2_-200H. Increasing reduction temperatures shifted Ti binding energies downward, reaching a maximum shift (458.4 eV) for Pt/TiO_2_-500H, before reverting to 458.7 eV at 600°C. Concurrently, Pt 4*f* binding energies decreased monotonically from 71.7 eV (Pt^δ⁺^) to 71.2 eV [Pt(0)], reflecting progressive electron enrichment in Pt as TiO_2−x_ coverage increased [33]. These results demonstrate that hydrogen reduction elevates electron density in both Ti and Pt via removal of interfacial oxygen in Ti–O–Pt linkages (Ti^4+^–O–Pt^δ⁺^ + H_2_ → Ti^3+^–Ov–Pt^0^ + H_2_O) (Fig. 3f) [21]. Defects generated at the TiO_2−x_/Pt interface, particularly in Pt/TiO_2_-500H, induced pronounced electron redistribution, underscoring the critical role of controlled reduction in tailoring interfacial electronic states.

### Photocatalytic properties and charge dynamics of the Pt/TiO_2_-*n*H photocatalysts

**Figure 4.**
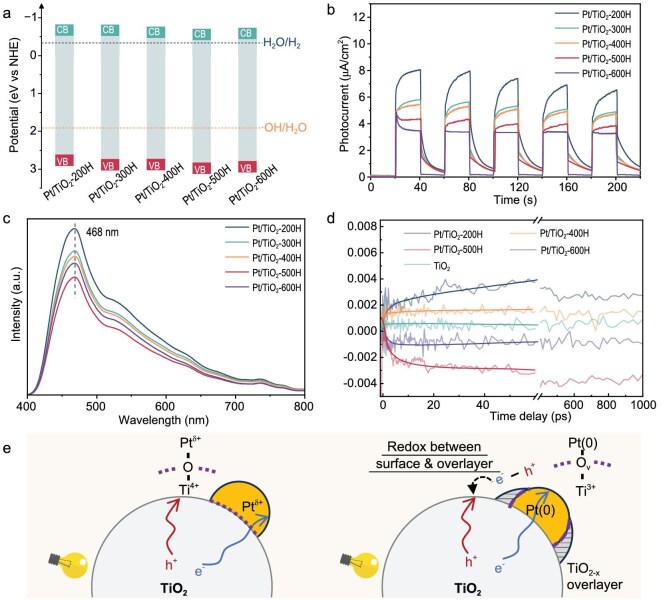
Optical properties of Pt/TiO_2_-*n*H catalysts. (a) Band structure of Pt/TiO_2_-*n*H catalysts determined by UV-vis spectra and Mott–Schottky plots. (b) Transient photocurrent profiles of Pt/TiO_2_-*n*H catalysts. (c) PL spectroscopy. (d) TAS. (e) Comparison of the transfer pathways of photoexcited carriers between fully exposed Pt/TiO_2_ (left) and Pt/TiO_2_ catalyst with TiO_2−x_ overlayer (right) (hole migration from TiO_2_ surface to TiO_2−x_ overlayer).

UV-vis absorption spectroscopy (Fig. S6) revealed that all Pt/TiO_2_ photocatalysts exhibited band gaps of approximately 3.20 eV (Table S1). Combined with flat band potentials obtained from Mott–Schottky measurements (Fig. S7), the calculated band structure (Fig. 4a) indicated that the hydrogen treatment does not influence the band structure of Pt/TiO_2_-*n*H catalysts significantly [34]. Photocurrent density measurements (Fig. 4b) showed a gradual decline with increasing reduction temperature, correlating with reduced light-harvesting efficiency as TiO_2−x_ coverage on the Pt cocatalyst increased. Photoluminescence (PL) spectra under 365 nm excitation (Fig. 4c) displayed emission signals near 468 nm, attributed to charge transfer from Ti^3+^ to oxygen anions in TiO_6_ octahedra associated with oxygen vacancies [35]. Such defects result in charge–carrier recombination, which is detrimental to photocatalytic activity. Notably, Pt/TiO_2_-500H with partially covered Pt and Ti^3+^–Ov–Pt^0^ interfacial structures, exhibited the lowest recombination probability, a finding corroborated by reduced charge-transfer resistance in electrochemical impedance spectra (EIS; Fig. S8).

Transient absorption spectroscopy (TAS) was employed to shed light on the ultrafast charge transfer process of photoexcited charge carriers in Pt/TiO_2_ catalysts and pristine anatase TiO_2_ experimentally. The transient signals at 455 nm from 0 ps to 1 ns corresponding to dynamics of photoholes revealed distinct trends (Fig. 4d) [36,37]. Compared to pristine TiO_2_, Pt/TiO_2_-200H and -400H exhibited prolonged hole signals due to the efficient electron transfer towards Pt co-catalysts. In contrast, the hole signals rapidly decreased after excitation on Pt/TiO_2_-500H (partially buried Pt) and -600H (fully covered Pt), where the TiO_2−x_/Pt inverse interface exists. Since Pt accepts electrons for all samples as shown in *in situ* Pt 4*f* XPS spectra, the drop of hole signals is unlikely to result from the recombination of electron-holes at such a fast timescale. Therefore, the declined hole signals are more likely attributed to the transfer of holes from the TiO_2_ support towards the overlayer sites of the TiO_2−x_/Pt inverse interface. Such a selective charge transfer of electrons to Pt and holes to the TiO_2−x_ overlayer at the inverse interface enables an efficient charge separation, which is favorable for the photocatalytic reactions. *In situ* Pt 4*f* XPS spectra (Fig. S9) under light radiation, showing a shift towards lower binding energy (71.2 to 70.8 eV), confirmed that Pt consistently functions as a photoelectron acceptor, independent of the encapsulation degree. This phenomenon underscores that the TiO_2−x_ overlayer, rich in Ti^3+^ sites (electron-enriched compared with the TiO_2_ surface), serves as the primary reservoir for photoinduced holes during the acetonitrile coupling reaction. Upon light radiation, the holes on the surface of the TiO_2_ support tend to oxidize the species in the electron-enriched TiO_2−x_ overlayer and induce hole migration from the typical TiO_2_ surface to the inverse TiO_2−x_/Pt structure (Fig. 4e). The migration of holes to the TiO_2−x_ surface facilitates radical generation (e.g. ·OH and ·CH_2_CN) at sites with the lowest *E*_ads_ as calculated by theoretical modeling, which can potentially accelerate their desorption into bulk solution.

### Photocatalytic dehydrogenative coupling of acetonitrile

**Figure 5.**
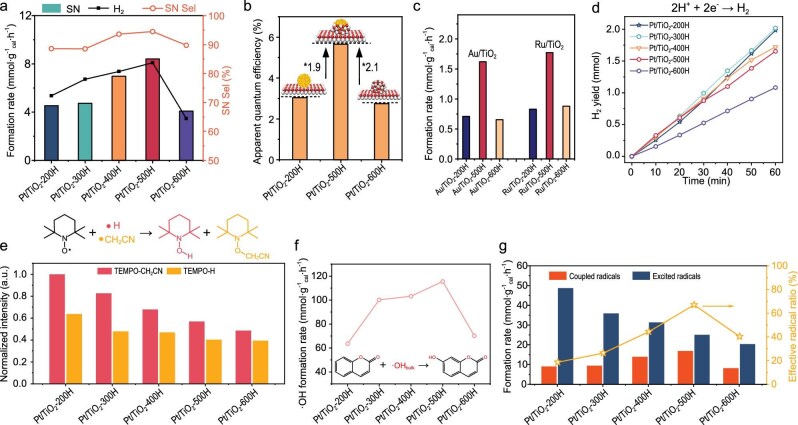
Catalytic performance evaluations of Pt/TiO_2_ photocatalysts and radical capturing experiment. (a) SN formation rates over Pt/TiO_2_-200H to Pt/TiO_2_-600H. (b) Apparent quantum efficiency of Pt/TiO_2_-200H, Pt/TiO_2_-500H and Pt/TiO_2_-600H. (c) SN formation rates over Au/TiO_2_ and Ru/TiO_2_. (d) Time course of H_2_ evolution on Pt/TiO_2_-200H to Pt/TiO_2_-600H during photocatalytic water-splitting using methanol scavenger. (e) Normalized intensity of free radicals captured by 0.5 g TEMPO in photocatalytic acetonitrile dehydrogenation coupling. (f) Fluorescence spectra of Pt/TiO_2_-200H to Pt/TiO_2_-600H deriving from the production of 7-OH-coumarin during photocatalysis experiment. (g) Formation rate of SN (coupled radicals) and ·CH_2_CN radicals captured by TEMPO (excited radicals), as well as the effective radical ratio.

The synthesized Pt/TiO_2_-*n*H catalysts were then evaluated for photocatalytic acetonitrile coupling reactions (Fig. 5a). All catalysts exhibited activity, with fully exposed Pt/TiO_2_-200H and fully encapsulated Pt/TiO_2_-600H showing ∼4 mmol · g_cat_^−1^·h^−1^ productivity and ∼90% selectivity to SN. However, neither achieved optimal activity. The activity and selectivity of the Pt/TiO_2_-*n*H series followed volcano-shaped trends with reduction temperature. Pt/TiO_2_-500H presents the peak SN formation rate of 8.41 mmol · g_cat_^−1^ · h^−1^ and 96% selectivity, suggesting the superiority of partial TiO_2−x_ encapsulation on Pt co-catalysts for enhancing acetonitrile coupling. Furthermore, Pt/TiO_2_-500H achieved an apparent quantum yield of 5.6%, which is 2.1- and 1.9-fold higher than the fully exposed and encapsulated counterparts (Fig. 5b), which demonstrates that optimizing the inverse interfacial structures is able to improve the light-to-chemical conversion efficiency significantly. Au/TiO_2_ and Ru/TiO_2_ catalysts treated in dilute H_2_ at 500°C also exhibited nearly 2-fold activity enhancements compared to their non-encapsulated and fully-encapsulated counterparts (Fig. 5c and Fig. S10). Therefore, this partial encapsulation can be a general strategy to promote the acetonitrile coupling performance.

To elucidate the role of the TiO_2−x_/Pt inverse interface in governing the catalytic performance of Pt/TiO_2_ catalysts in acetonitrile coupling, we quantified the rates of key steps in the photocatalytic dimerization reaction. Previous mechanistic studies indicate that photocatalytic acetonitrile coupling proceeds via a dehydrogenative pathway, with water acting as a co-catalyst. The reaction initiates with water oxidation by photoinduced holes, generating hydroxyl radicals (·OH) and protons (H⁺). Protons are subsequently reduced by photoexcited electrons on the Pt cocatalyst surface, releasing H_2_ gas. ·OH radicals selectively abstract hydrogen from the C_α_ atom of acetonitrile (CH_3_CN) to form ·CH_2_CN radicals as the key intermediate species. SN, the final C–C coupling product, arises from homo-termination of two ·CH_2_CN radicals [6]. Thus, the overall efficiency hinges on four critical steps, namely: (i) hydrogen evolution; (ii) radical initiation; (iii) radical desorption; and (iv) radical coupling.

Hydrogen evolution rates of Pt/TiO_2_-*n*H catalysts, probed via photocatalytic water splitting with methanol as a sacrificial agent, decreased from 2.0 to 1.0 mmol · h^−1^ as reduction temperatures rose (Fig. 5d), correlating with diminishing exposed Pt surface area. This trend aligns with photocurrent data (Fig. 4b) and confirms that H_2_ evolution is sensitive to Pt accessibility. It also confirms that hydrogen evolution is not the rate-limiting step in acetonitrile coupling.

The ability of radical initiation of Pt/TiO_2_-*n*H photocatalysts was determined using the radical capture method with excessive 2,2,6,6-tetramethylpiperidinyl-1-oxide (TEMPO) as the trapping reagent (Fig. 5e and Fig. S11). Surprisingly, the gas chromatography (GC) quantification results show that the formation rates of TEMPO-H (retention time = 12.8 min) and TEMPO-CH_2_CN (retention time = 14.9 min) both decrease with the elevating reduction temperature, indicating that the initiated hydrogen and ·CH_2_CN radicals in unit time are both suppressed by the formation of the TiO_2−x_ overlayer. Compared with the apparent activity of the photocatalysts, it can be concluded that not all the stimulated ·CH_2_CN radicals enter the final products, especially on the photocatalysts with a fully exposed Pt cocatalyst.

A fluorescence probe method using the hydroxyl-induced coumarin oxidation reaction was applied to detect desorbed ·OH from the surface of the heterogeneous photocatalyst into the aqueous suspension (Fig. 5f) [38,39]. The fluorescence intensity of the hydroxyl-functionalized coumarin increased first with the elevating TiO_2−x_ coverage on Pt NPs and reached a maximum at 500°C. Further increasing the TiO_2−x_ surface coverage causes a reduction in the release rate of hydroxyl radicals. This trend also confirms the DFT calculation results, which predicted that the desorption of ·OH from the inverse interface is easier than from the TiO_2_ surface. The concentration of hydroxyl species in the aqueous solution showed a good correlation with the apparent activity of Pt/TiO_2_-*n*H in the coupling reaction, suggesting the activity is probably determined by the radicals escaping from the surface of the catalysts. The rate of ·CH_2_CN radical coupling into SN can be calculated from product distribution, which confirms that about 70% of the ·CH_2_CN radicals excited on the Pt/TiO_2_-500H are converted into SN (Fig. 5g). In contrast, even though the fully exposed Pt co-catalyst (Pt/TiO_2_-200H) induces the highest ·CH_2_CN initiation rate, only <20% of the ·CH_2_CN radicals manage to convert into product. Since no other carbon-containing products were observed, we conclude that the excess ·CH_2_CN radicals primarily undergo reverse reaction with protons and photoelectrons to regenerate acetonitrile. To directly verify this hypothesis, we conducted deuterium (D)-labeling experiments by replacing H_2_O with D_2_O (Fig. S12). The clear detection of deuterated acetonitrile (CDH_2_CN/CD_2_HCN) in the products provides unequivocal evidence that ·CH_2_CN radicals are indeed quenched by protons/deuterons to revert to acetonitrile. Most critically, the formation of deuterated acetonitrile was most pronounced over the fully exposed Pt/TiO_2_-200H catalyst, while being significantly suppressed over the optimally encapsulated Pt/TiO_2_-500H. These D-labeling results offer direct experimental validation of our proposed mechanism, effectively complementing the DFT calculations and product analysis, and firmly establish the critical role of the TiO_2−x_ overlayer in mitigating radical–proton recombination. The good correlation of the effective radical efficiency with the surface coverage of the TiO_2−x_ overlayer suggests that the formation of inverse TiO_2−x_/Pt interfacial sites indeed suppresses the undesirable reverse surface reaction. This pronounced promotion is due to the migration of excited holes from the TiO_2_ surface to the TiO_2−x_ overlayer of the inverse structure, where the *E*_ads_ of ·OH and ·CH_2_CN radicals is significantly reduced due to the interfacial charge redistribution and the abundant Ti^3+^–Ov–Pt(0) sites formed at the inverse interface. The partial covering of the Pt surface also reduces the possibility of radical quenching at the metal surface where stimulated electrons are located. The quantitative analysis of the reaction kinetics also demonstrates that suppressing the undesirable reverse reaction of the excited radical intermediate is more important to promote the photocatalytic acetonitrile coupling efficiency than enhancing the radical initiation capability. Constructing an inverse encapsulation structure with appropriate coverage using a TiO_2−x_ overlayer emerges as an efficient strategy to achieve this target. Long-term stability tests were conducted over 12 h using the Pt/TiO_2_-500H and Pt/TiO_2_-200H catalysts (Fig. S13). The results demonstrate that Pt/TiO_2_-500H exhibits excellent durability, with the SN formation rate remaining nearly constant and only a slight decrease in catalytic activity. In contrast, Pt/TiO_2_-200H showed noticeable deactivation. The enhanced stability of Pt/TiO_2_-500H is attributed to the partially encapsulated TiO_2−x_ overlayer, which effectively anchors Pt NPs and prevents agglomeration or leaching. These results confirm the robustness of the TiO_2−x_/Pt inverse heterostructure and strongly support the proposed reaction mechanism.

## CONCLUSIONS

In summary, we demonstrate that a subsurface Pt-stabilized TiO_2−x_ overlayer synthesized via high-temperature H_2_ reduction, predicted by DFT calculation, significantly enhances both selectivity and productivity in the photocatalytic dehydrogenative C–C coupling of acetonitrile to SN. The TiO_2−x_ overlayer serves as a reservoir for photoinduced holes with low *E*_ads_ for reactive intermediates (·OH and ·CH_2_CN radicals). Compared to conventional fully exposed Pt/TiO_2_ catalysts, the partially encapsulated Pt/TiO_2−x_ system exhibits a 3-fold increase in effective radical utilization efficiency, doubling SN productivity (8.41 vs. 4.00 mmol · g_cat_^−1^·h^−1^) and improving selectivity by ∼10% (96% vs. 86%). Kinetic studies reveal that suppressing undesirable reverse reactions of radical intermediates, achieved by weakening radical adsorption through interfacial charge redistribution at the inverse interface, is pivotal to optimizing photocatalytic performance. This work establishes a universal/compatible strategy for enhancing photocatalytic C–C coupling reactions: engineering inverse metal–support interfaces with controlled oxide overlayers to modulate radical dynamics and minimize recombination.

## METHODS

### Photocatalytic dehydrocoupling of acetonitrile

Photocatalytic experiments (acetonitrile dehydrogenative coupling to SN) were performed in a top-irradiation Pyrex flask. A 10 W LED light (wavelength 365 nm) (PLS-SXE 300, Beijing Trusttech Co., Ltd.) was used as the light source. Typically, 20 mg of photocatalysts were dispersed in 10 mL of 70% volume acetonitrile aqueous solution under magnetic stirring. Prior to the irradiation, the reaction mixture was deaerated repeatedly with Ar gas five times to remove air and dissolved oxygen thoroughly. During the reaction, the photocatalytic reaction system was kept at 60°C. To evaluate the photocatalytic hydrogen production and analyze other gas products, the gas-phase composition of the photocatalytic reactor was analyzed by an Agilent 8860 gas chromatograph equipped with 5 Å molecular sieves and HP-Plot columns and a thermal conductivity cell detector (TCD). Liquid products were analyzed by an Agilent 8860 gas chromatograph equipped with a column of SH-1 with a flame ionization detector (FID).

### Radical capturing experiments

The ·OH radical intermediates in the photocatalytic dehydrocoupling of acetonitrile were trapped by coumarin. Briefly, 10 mg of photocatalyst powder was dispersed in an acetonitrile aqueous solution containing 10 mL of 0.1 mM coumarin. The solution was irradiated at 60°C and stirred for 20 min with an LED lamp (365 nm). After irradiation, the supernatant was determined by a fluorescence spectrophotometer (Fls 1000), with the excitation wavelength of 332 nm.

The ·CH_2_CN radical intermediates in the photocatalytic dehydrocoupling of acetonitrile were trapped by TEMPO. Briefly, an additional 1 g of TEMPO was put into the routine reaction system; the remaining conditions and steps are consistent with the photocatalytic reaction evaluation described in the Supplementary data. The products were analyzed using an Agilent 8860 gas chromatograph equipped with an HP-5 column and FID.

### DFT calculations

All spin-polarized calculations were carried out with Vienna *Ab Initio* Simulation Package (VASP) [23], with the frozen-core projected-augmented wave (PAW) method [22] being used with application of the generalized gradient approximation of Perdew–Burke–Ernzerhof (PBE) [40]. PBE plus U with a *U*_eff_ of 4.0 eV was used to describe the Ti 3*d* electronic states to correct for the on-site Coulomb interactions [18]. A plane wave cutoff of 450 eV was used to describe the electronic wave function. The criterion for self-consistent iterations is that the charge in energy between successive steps is converged to 1.0 × 10^−5^ eV, and the forces on each atom are converged to 0.02 eV/Å. A gamma-centered grid of (1 × 1 × 1) k-points was used for Pt-NPs/TiO_2_(101) and (2 × 2 × 1) for other models.

In the TiO_2_(101) model, a periodical (3 × 3) supercell with two O–Ti–O layers was built from the bulk phase; it contains 36 Ti and 72 O atoms. For Pt-NPs/TiO_2_(101), there are 72 Ti, 144 O and 31 Pt atoms. During the calculations, the bottom half of the atoms were fixed. The Pt(111) model contains a (4 × 4) supercell with four atomic layers. The bottom two layers were fixed, and the top two layers were allowed to relax during all calculations. For the heterojunction TiO_2_(101)/Pt(111) model, a p(2 × 3) TiO_2_(101) layer was loaded on a three-layer p(4 × 4) Pt(111) slab; there are 48 Pt, 12 Ti and 24 O atoms, during the optimization. For the 0.5 monolayer TiO_2_(101)/Pt(111), there are 48 Pt, 6 Ti and 12 O atoms; the bottom two Pt layers were fixed and others were allowed to relax.

## Supplementary Material

nwaf588_Supplemental_File
