# Supplementary material for: The subsurface Pt-promoted TiO2−x overlayer enhances succinonitrile production in the photocatalytic C–C coupling of acetonitrile
Source: Natl Sci Rev. 2025 Dec 27;13(4):nwaf588. doi: 10.1093/nsr/nwaf588 (PMC12900415; doi:10.1093/nsr/nwaf588)
Supplement: nwaf588_Supplemental_File [file nwaf588_supplemental_file.pdf]

Supporting Information

The subsurface Pt-promoted TiO_{2-x} overlayer enhances succinonitrile production in the photocatalytic C-C coupling of acetonitrile

Xian Zhou,[a+] Houhong Song,[a+] Zhitong Chen,[a] Shufang Zhao,[c] Junting Wang,[d] Yiou Wang,[d] Xiaofeng Gao,[a,b*] Lili Lin,[c*] and Siyu Yao,[a,b*]

[a] Key Laboratory of Biomass Chemical Engineering of Ministry of Education, College of Chemical and Biological Engineering, Zhejiang University, Hangzhou 310058, China.

[b] Institute of Zhejiang University-Quzhou, Quzhou 324000, China.

[c] Institute of Industrial Catalysis, State Key Laboratory of Green Chemistry Synthesis Technology, College of Chemical Engineering, Zhejiang University of Technology, Hangzhou 310014, China.

[d] School of Interdisciplinary Science, Beijing Institute of Technology, Beijing, 102488, P. R. China

Table of Content

Experimental Section 3

Figure S1. Projected density of states (pDOS). 7

Figure S2. The corresponding EDX mapping of Pt/TiO_2-200H catalyst. 8

Figure S3. The corresponding EDX mapping of Pt/TiO_2-500H catalyst. 9

Figure S4. The corresponding EDX mapping of Pt/TiO_2-600H catalyst. 10

Figure S5. O 1s XPS spectra of Pt/TiO_2-nH photocatalysts. 11

Figure S6. UV-Vis diffuse reflectance spectra. 12

Figure S7. The Mott-Schottky plots and corresponding flat band potential. 13

Figure S8. The electrochemical impedance spectroscopy. 14

Figure S9. XPS spectra of Pt/TiO_2-500H before and after illumination. 15

Figure S10. High-magnification STEM-ADF images of Au/TiO_2-200H. 16

Figure S11. GC-MS spectra of the radical capturing experiment using TEMPO. 17

Figure S12. The deuterium content ratio obtained through mass spectrometry analysis. 18

Figure S13. Time dependent catalytic performance. 19

Table S1. ICP, size distribution, band gap and CO desorption results. 20

Table S2. Catalytic performance of of Pt/TiO_2-nH photocatalysts. 21

References 22

Experimental Section

2.1 Materials.

All chemicals were used directly without further purification. Anatase TiO_2 was purchased from Aladdin. $H_2PtCl_6 6H_2O$, $HAuCl_4 4H_2O$, and $RuCl_3$ were purchased from Macklin.

2.2 Catalyst Preparation.

The M/TiO_2 catalysts were first prepared by photodeposition method.[1] In the photodeposition (PD) of metal over TiO_2 support, 200 mg TiO_2 powder and metal precursor ($H_2PtCl_6{\cdot}6H_2O$ / $HAuCl_4{\cdot}4H_2O$ / $RuCl_3$) were dispersed in 15 mL 20% methanol aqueous solution (v/v) in a quartz flask. Subsequently, the flask was placed in the 365 nm photo-radiation generated by a 1W LED lamp under argon protection with vigorous stirring for 3 h. After the PD procedure, the M/TiO_2 was separated from the suspension by centrifugation, washed 3 times using deionized water and dried at 333 K for 12 h. The sample was then reduced in H_2 at 200-600 °C for 2 h. The resulting photocatalysts were denoted as Pt/TiO_2-nH, where n represents the hydrogen treatment temperature (°C).

2.3 Sample Characterizations.

2.3.1 ICP-AES

The loadings of supported metals on the catalysts were analyzed by inductively coupled plasma atomic emission spectrometry (ICP-AES) on a Varian ICP-OES 720 instrument. In a typical procedure, 25mg catalyst powder was dissolved by 3 mL HF and 4 mL chlorazotic acid. The solution as filtrated and diluted by DI water for further measurement.

2.3.2 X-ray Diffraction characterization

Powder X-ray diffraction (XRD) patterns were recorded in the 2θ range 10°-80° on a Philips X'Pert Pro Super diffractometer with Cu Kα radiation (λ = 0.15406 nm) operating at 40 kV and 50 mA.

2.3.3 Transmission electron microscopy (TEM)

The TEM and HADDF-STEM images of catalysts were performed with a JEOL JEM-2100F instrument at an acceleration voltage of 200 kV.

2.3.4 Electron paramagnetic resonance (EPR) spectra

Electron paramagnetic resonance (EPR) spectra were recorded on a JEOL JES-FA200 EPR spectrometer (9.063 GHz, X-band) at 130 K with employed microwave power, modulation frequency, and modulation amplitude of 0.998 mW, 100 kHz, and 0.35 mT, respectively.

2.3.5 UV-Vis Spectroscopic characterization

UV-vis diffuse reflectance spectra (UV-vis DRS) were obtained on a Shimadzu DUV-3700 spectrophotometer equipped with an integrating sphere attachment.

2.3.6 Transient photocurrent

The 5 mg sample was mixed with 0.5 ml ethanol solution for ultrasonic treatment for 1 hour, and then the prepared sample was dropped onto ITO conductive glass of 1 cm × 2 cm. The sample was dried in the oven at 60°C to ensure that the sample would not fall off. The glass was used as

the working electrode. The electrolyte solution was 1 M Na_2SO_4 solution with reference electrode of mercurous sulfate and counter electrode of Pt. The electrochemical workstation was manufactured by Shanghai Chenhua Instrument Co., LTD. The test method was i-t Curve with a sensitivity of 1.e-6. During the test, the conductive glass stained with the sample was inserted into the electrolyte solution and fixed in a suitable position. Then turn on the light (with a 365nm filter) and test for 20s. Repeat the above steps 5 times to obtain the photocurrent spectrum.

2.3.7 Photoluminescence spectroscopy
Horibalabram-hr confocal laser microraman spectrometer produced by JY, France, was used for PL spectrum. The excitation light source was HE-Cd laser (365nm) and the grating was 2400.

2.3.8 Electrochemical impedance spectroscopy and Mott–Schottky curves
Electrochemical impedance spectroscopy (EIS) and Mott–Schottky curves weremeasured on electrochemical workstation manufactured by ShanghaiChenhua Instrument Co. LTD. Typically, 5 mg sample was mixed with 1 mL ethanol aqueous solution (volume fraction 30 %) and 30 μL nafion-117 solution (~5 % in a mixture of lower aliphatic alcohols and water from Aladdin) for ultrasonic treatment for 1 hour, and then the prepared sample was dropped onto polished and activated glassy carbon electrode (GCE). The sample was dried in the oven at 60 °C to ensure that the sample would not fall off. The GEC was used as the working electrode. The electrolyte solution was 0.1 M Na_2SO_4 solution with reference electrode of saturated silver chloride electrode and counter electrode of Pt. The test method was A.C. impedance (IMP), I-t Curve (It) and impedance-potential (IMPE) respectively.

2.3.9 Fluorescence probe experiment
Photocatalyst powder (10 mg) was suspended in a Pyrex cell containing 10 mL of a 0.1 mM coumarin aqueous-CH_3CN solution, and one side of the cell was irradiated with the LED beam. The suspension was stirred vigorously for 20 min before and during the light irradiation at 60 °C. After the irradiation, the clear solution was taken out and its fluorescence spectrum was measured by the Fls1000 fluorescence spectrophotometer with the excitation wavelength at 332 nm.

2.3.10 Radical capturing experiments
The radical capturing experiments were conducted using 2,2,6,6-tetramethylpiperidoxyl (TEMPO) as the trapping reagent. An additional 1 g of TEMPO is routinely put into reaction system, the remaining conditions and steps are consistent with the photocatalytic reaction evaluation described above. The products were analyzed using an Agilent 8860 gas chromatograph equipped with an HP-5 column and flame ionization detector (FID).

2.3.11 Transient absorption spectroscopy
Transient absorption spectroscopy was used to measure the charge carrier behavior in the photocatalytic process. The measurement was performed under ambient conditions using a femtosecond amplifier laser system (140 fs, 1000 Hz, 800 nm, Coherent Evolution). A short laser pulse was generated and split into two beams. One beam generated a 355 nm pump pulse through optical parametric amplification, which was focused onto the sample to excite charge carriers optically. The other part of the initial pulse generated the white light spectrum as the probe pulse. And the probe and pump pulses overlapped spatially in the sample through a time

delay stage. Ultimately, the probe light was collected by the monochromator (Acton SP 2500, Princeton Instruments, USA), and the signal was recorded by a charge-coupled device (CCD) camera. Samples were dispersed in ethanol, uniformly coated on a quartz wafer, and measured after the evaporation of ethanol. First, 10 mg of the sample was dispersed in ethanol and fully sonicated, and then the small particles were uniformly coated on a quartz wafer using a spin coater. Finally, the sample was measured with a pump light power of 21.9 μw under ambient conditions.

2.4 Photocatalytic Reaction Test.

Photocatalytic experiments (acetonitrile dehydrogenative coupling to succinonitrile) were performed in a top-irradiation Pyrex flask. A 10W LED light (wavelength 365 nm) (PLS-SXE300, Beijing Trusttech Co., Ltd.) was used as the light source. Typically, 20 mg photocatalysts were dispersed in 10 mL 70% volume acetonitrile aqueous solution under magnetic stirring. Prior to the irradiation, the reaction mixture was deaerated repeatedly with Ar gas for 5 times to thoroughly remove air and dissolved oxygen. During the reaction, the photocatalytic reaction system was kept at 60°C. To evaluate the photocatalytic hydrogen production and analyze other gas products, the gas-phase composition of the photocatalytic reactor was analyzed by an Agilent 8860 gas chromatograph equipped with 5 Å molecular sieves and HP-Plot columns and thermal conductivity cell (TCD) detector. Liquid products were analyzed by Agilent 8860 gas chromatograph equipped with a column of SH-1 with flame ionization detector (FID). The formation rate of all products and carbon-based selectivity of products were defined in equation (1) - (2).

Formation rate

$$r=\frac{n_x}{t\times m_{cat}}$$

Eq (1)

Carbon based selectivity of products

$$S_x=\frac{a\times n_x}{4\times n_{SN}+n_{CO2}+2\times n_{AM}}$$ (a: The number of C atoms in x molecule) Eq (2)

2.5 **AQY calculation methods.**

In generally, producing one $\cdot CH_2CN$ radical from CH_3CN needs one hole with one electron consumed at the same time (e.g., one SN molecule needs two electrons). Then the AQY could be calculated according to the following Equation:

$$AQY(\%)=\frac{\text{number of reacted electrons}}{\text{number of incident photons}}\times 100\%$$

$$=\frac{\text{number of evolved SN molecules}\times 2}{\text{number of incident photons}}\times 100\%$$

The total light intensity incident (305 mW/cm^2) at the sample position is measured by using CEL-NP 2000-2A flux meter. Assuming uniform intensity distribution of the lamp, a correction for the difference in the area of the sensor of lux meter and the reactor surface area is evaluated.

AQY calculation:

Number of incident photons:

$$N = \frac{I \times A \times \lambda \times t}{h \times c} = \frac{3.05\times10^{3}\times1.77\times10^{-4}\times365\times10^{-9}\times3600}{6.626\times10^{-34}\times3\times10^{8}} = 3.57\times10^{21}$$

I: optical power density ($W\cdot m^{-2}$)
A: incident illumination area (m^2)
λ : wavelength of incident light (m)
t: time (s)
H: Planck constant (6.626×10^{-34} J·s)
C: the speed of light (3×10^{8} $m\cdot s^{-1}$)

$$AQY(\%) = \frac{\text{number of evolved SN molecules}\times 2}{N}\times100\%$$

2.6 DFT.

Calculation details:

All spin-polarized calculations were carried out with Vienna Ab-Initio Simulation Package (VASP)[2] with the frozen-core projected-augmented wave (PAW) method[3] was used with application of the generalized gradient approximation of Perdew-Burke-Ernzerhof (PBE).[4, 5] PBE plus U with a Ueff of 4.0 eV was used to describe the Ti 3d electronic states to correct for the on-site Coulomb interactions.[6] A plane wave cutoff of 450 eV was used to describe the electronic wave function. The criterion for self-consistent iterations is that the charge in energy between successive steps is converged to 1.0 × 10−5 eV, and the forces on each atom are converged to 0.02 eV/Å. A Gamma-centered grid of (1×1×1) k-points was used for Pt-NPs/TiO_2(101) and (2×2×1) for other models.

Calculation models:

In TiO_2(101) model, a periodical (3×3) supercell with two O-Ti-O layers was built from the bulk phase, it contains 36 Ti and 72 O atoms. For Pt-NPs/TiO2(101), there are 72 Ti, 144 O and 31 Pt atoms. During the calculations, the bottom half atoms were fixed. The Pt(111) model contains (4×4) supercell with four atomic layers. The bottom two layers were fixed and the top two layers were allowed to relax during all calculations. For heterojunction TiO_2(101)/Pt(111) model, a p(2×3) TiO_2(101) layer was loaded on a three-layer p(4×4) Pt(111) slab, there are 48 Pt, 12 Ti and 24 O atoms, during the optimization, for 0.5 monolayer TiO_2(101)/Pt(111), there are 48 Pt, 6 Ti and 12 O, the bottom two Pt layers were fixed and others were allowed to relax.

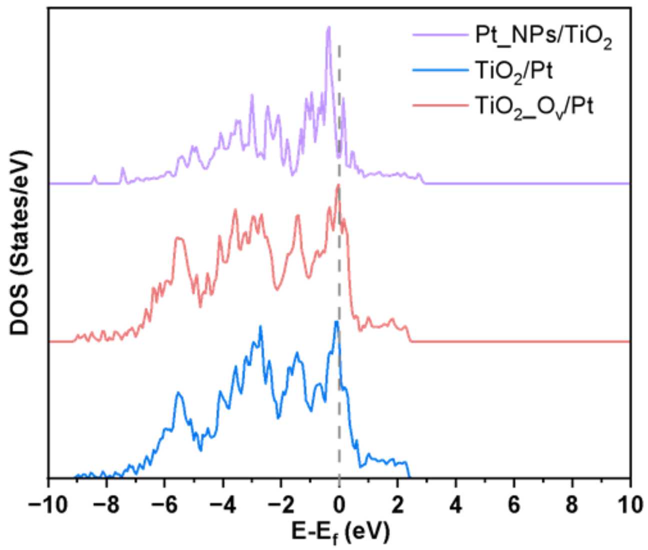

Figure S1. Projected density of states (PDOS) for Pt_NPs/TiO_2, TiO_2_O_v/Pt and TiO_2/Pt.

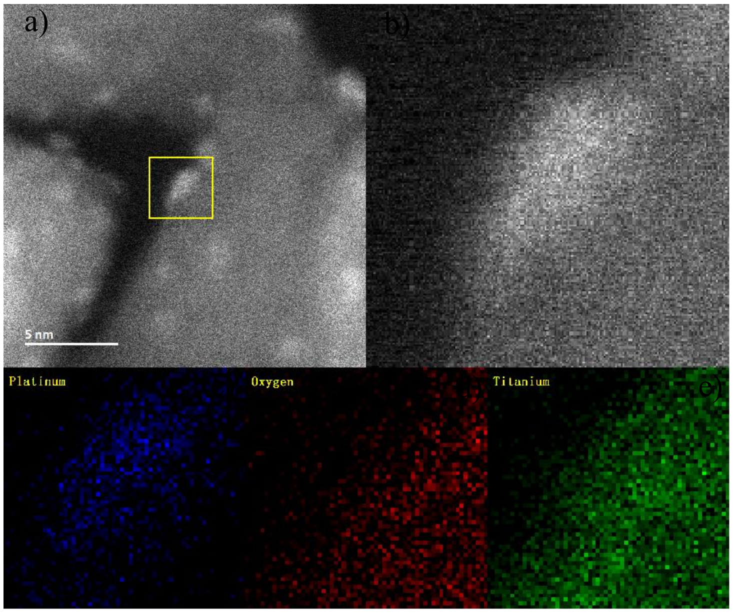

Figure S2. The corresponding EDX mapping of Pt, O, and Ti elements of Pt/TiO_2-200H catalyst. The yellow square in (a) highlights the selected region for elemental mapping analysis.

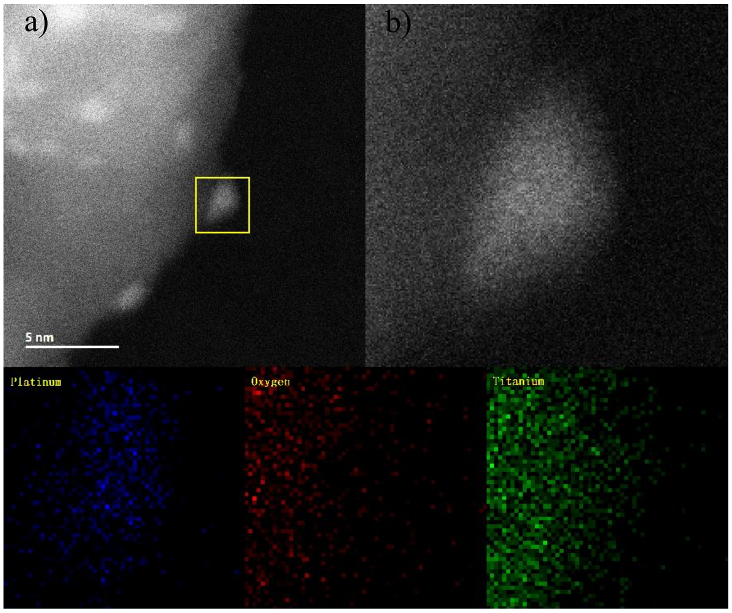

Figure S3. The corresponding EDX mapping of Pt, O, and Ti elements of Pt/TiO_2-500H catalyst. The yellow square in (a) highlights the selected region for elemental mapping analysis.

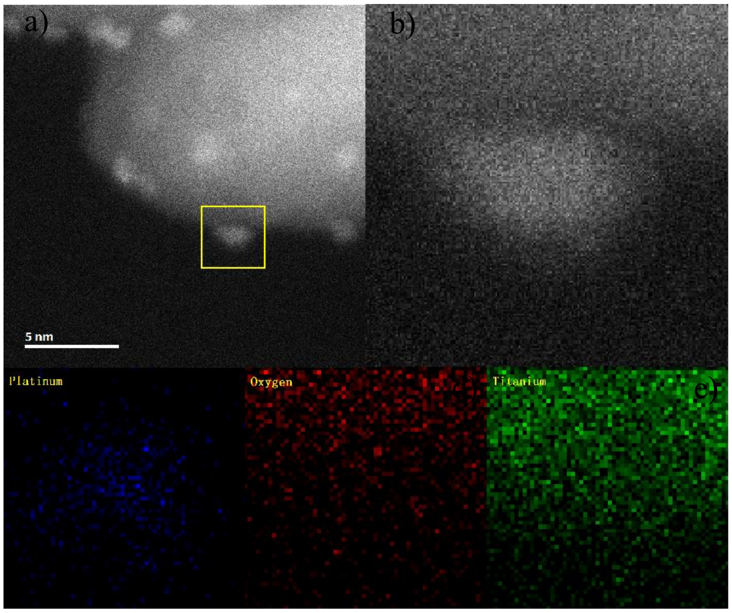

Figure S4. The corresponding EDX mapping of Pt, O, and Ti elements of Pt/TiO_2-600H catalyst. The yellow square in (a) highlights the selected region for elemental mapping analysis.

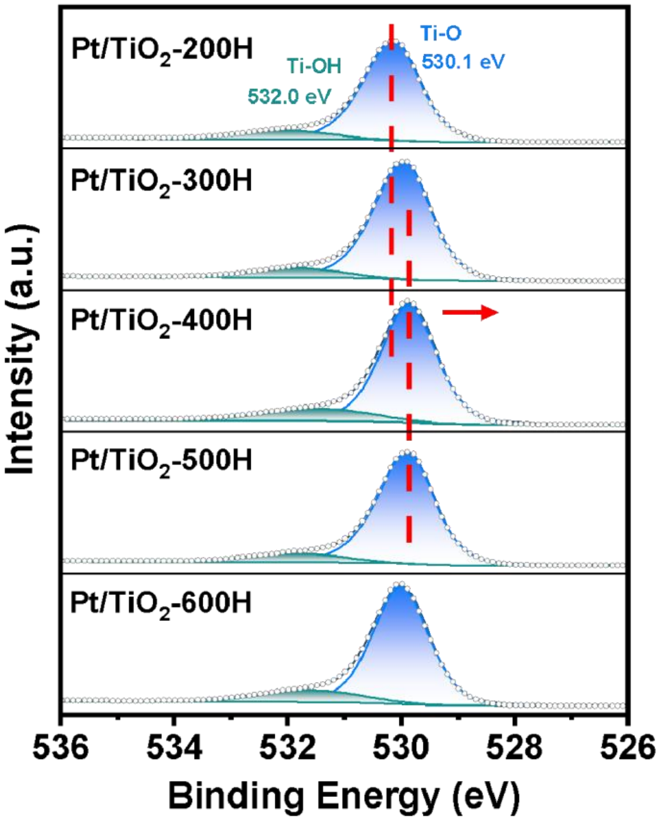

Figure S5. O 1s XPS spectra of Pt/TiO_2-nH photocatalysts.

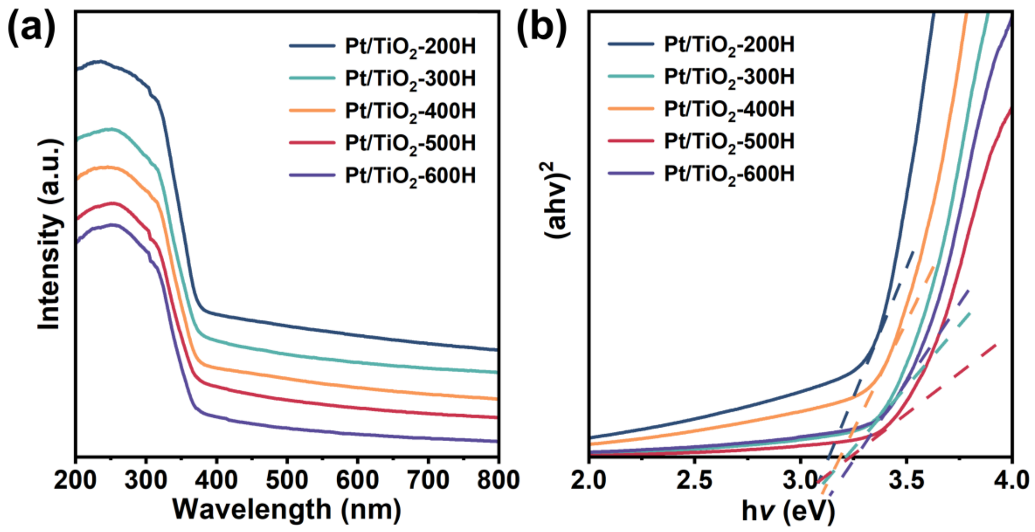

Figure S6. (a) UV-Vis diffuse reflectance spectra of Pt/TiO_2-nH photocatalysts; (b) The plots of modified Kubelka-Munk function versus the photon energy of Pt/TiO_2-nH photocatalysts.

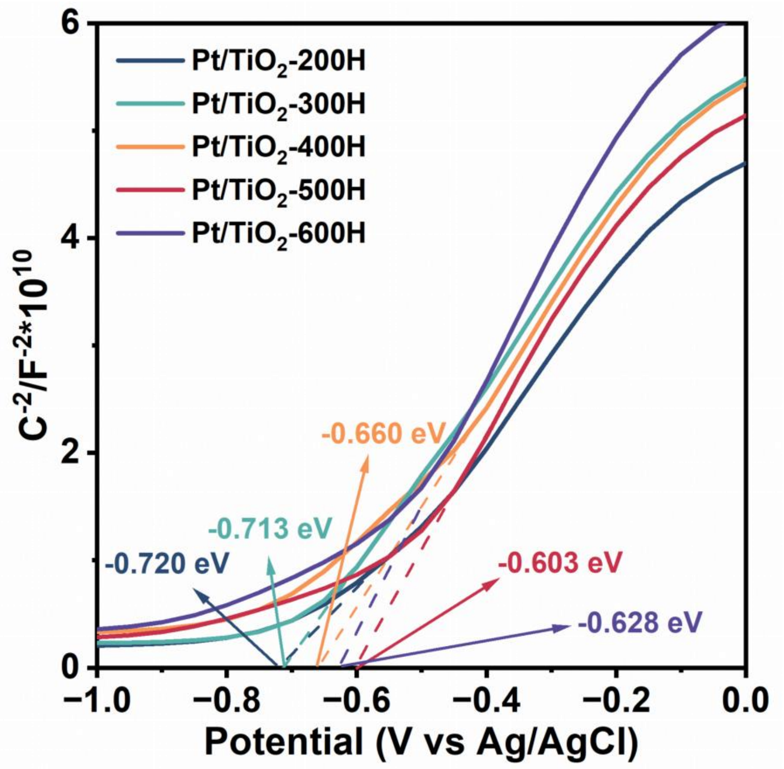

Figure S7. The Mott-Schottky plots and corresponding flat band potentialof Pt/TiO_2-nH photocatalysts.

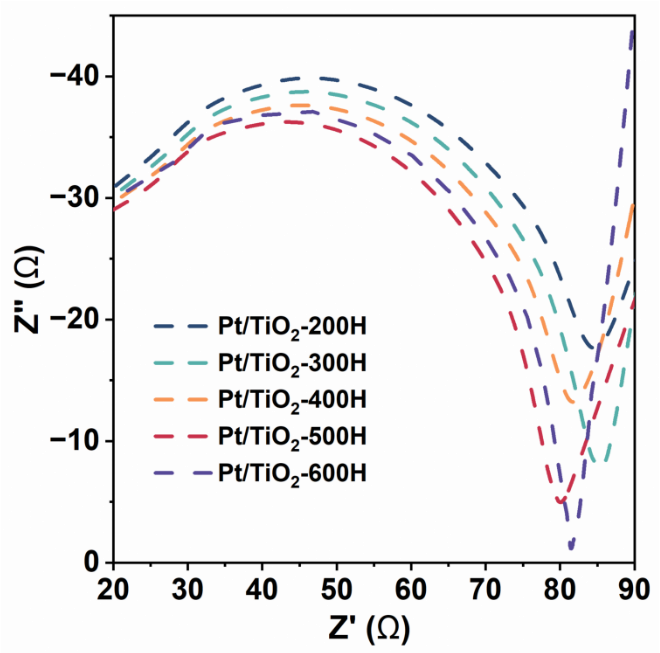

Figure S8. The electrochemical impedance spectroscopyof Pt/TiO_2-nH photocatalysts.

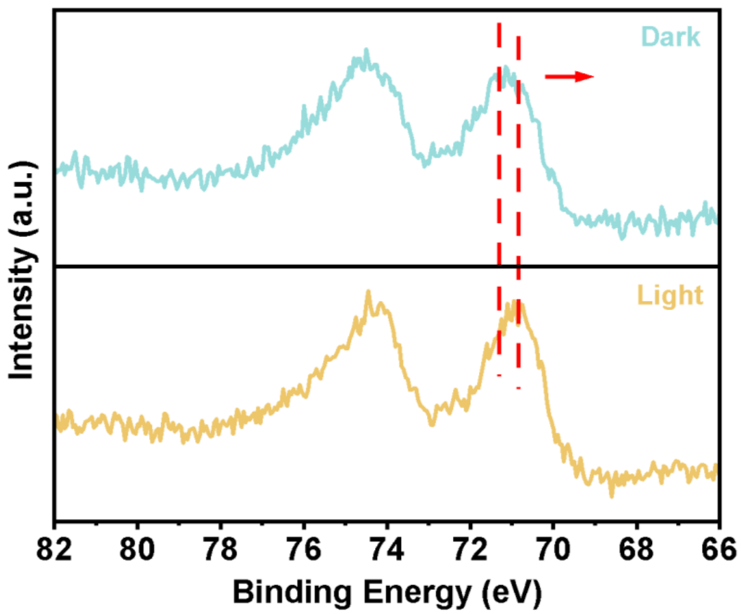

Figure S9. XPS spectra of Pt/TiO_2-500H before and after illumination.

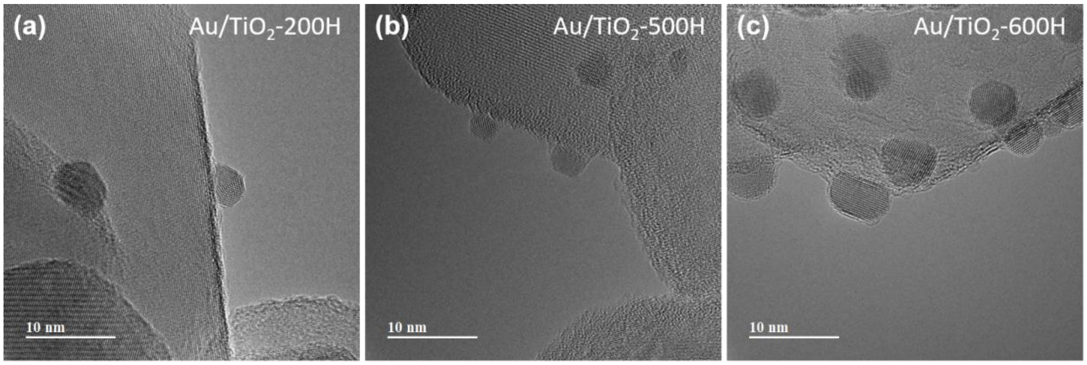

Figure S10. High-magnification STEM-ADF images of (a) Au/TiO_2-200H; (b) Au /TiO_2-500H; (c) Au /TiO_2-600H.

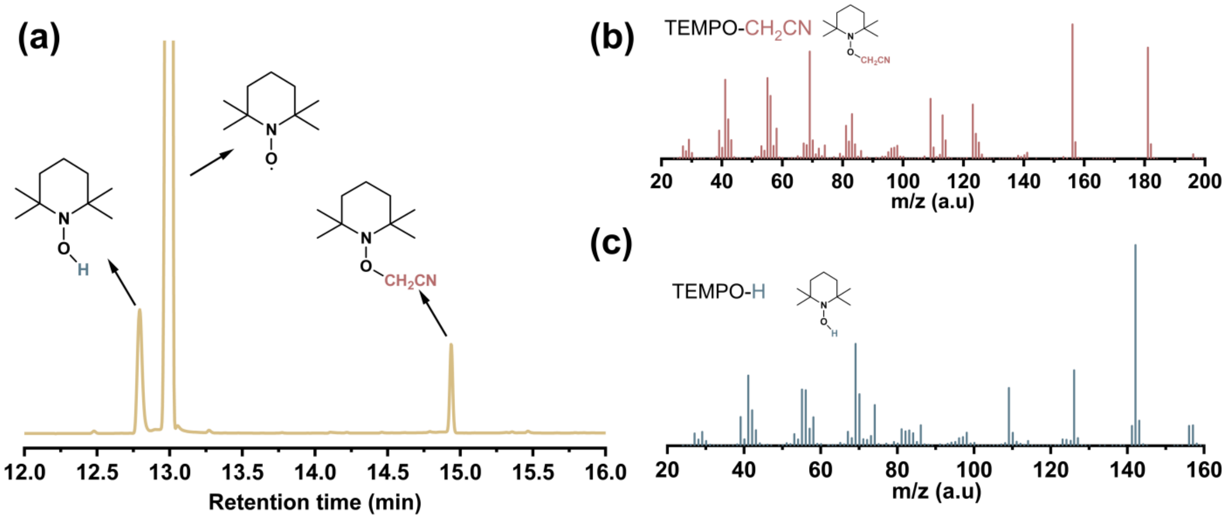

Figure S11. (a) GC-MS spectra of the radical capturing experiment using TEMPO; (b) Mass spectrogram of acetonitrile radical product captured by TEMPO and (c) hydrogen radical product captured by TEMPO.

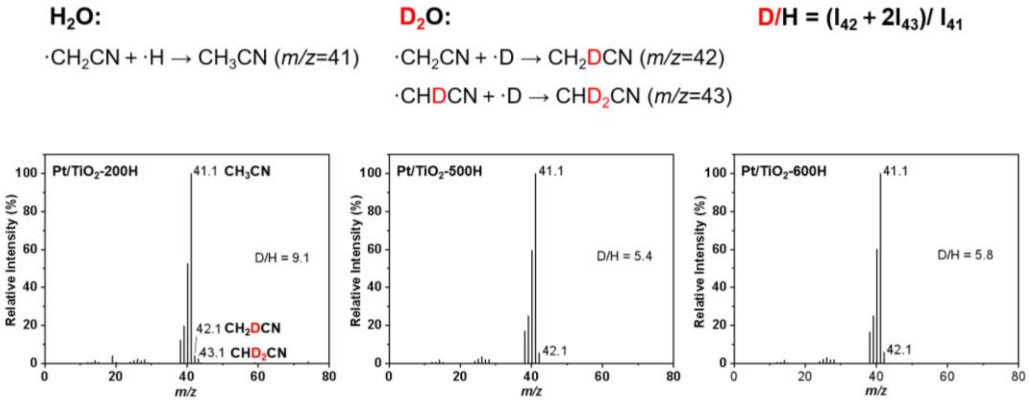

Figure S12. The deuterium content ratio obtained through mass spectrometry analysis.

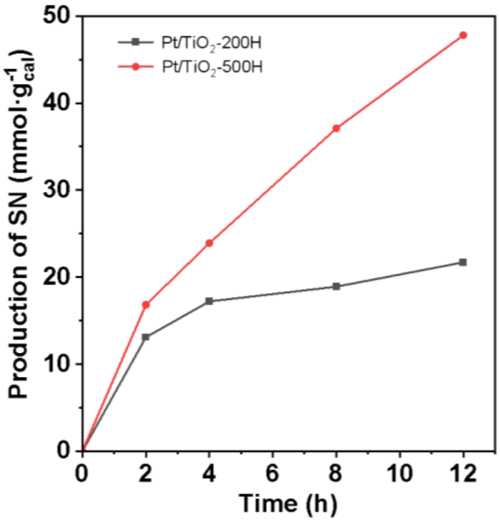

Figure S13. Time dependent catalytic performance.

Table S1. ICP, size distribution, band gap and CO desorption results of Pt/TiO_2-nH photocatalysts

Catalysts	Pt loading	Size distribution	Band gap	Band gap	Pt dispersion
200H	1.4 %	1.5 ± 0.3 nm	3.14 eV	3.14 eV	43.5 %
300H	1.3 %	1.5 ± 0.6 nm	3.25 eV	3.25 eV	33.1 %
400H	1.5 %	1.5 ± 0.5 nm	3.19 eV	3.19 eV	30.0 %
500H	1.5 %	1.4 ± 0.4 nm	3.24 eV	3.24 eV	13.7 %
600H	1.6 %	1.7 ± 0.5 nm	3.24 eV	3.24 eV	4.5 %

Table S2. Catalytic performance of of Pt/TiO_2-nH photocatalysts

Catalyst [a]	Formation rate (mmol g_{cat}^{-1} h^{-1})				Carbon based Selectivity (%) [b]			AQY (%)
	SN [d]	H_2	CO_2	AM [e]	SN	CO_2	AM	
Pt/TiO_2-200H	4.53	5.34	1.03	0.65	88.6	5.0	6.4	3.0
Pt/TiO_2-300H	4.73	6.70	0.93	0.77	88.5	4.4	7.1	3.1
Pt/TiO_2-400H	6.97	7.34	0.59	0.66	93.6	2.0	4.4	4.6
Pt/TiO_2-500H	8.41	8.06	0.59	0.69	94.5	1.7	3.8	5.6
Pt/TiO_2-600H	4.09	3.44	0.59	0.63	89.8	3.2	7	2.7

a. Reaction condition: Solution: 7mL CH_3CN + 3mL H_2O; Atmosphere: Ar; Light source: 10W LED lamp; UV-Vis light, λ = 365 nm; Irradiation time: 2 h

b. Carbon based selectivity is determined by GC.

c. Turnover frequency of the catalyst is defined as the average formation rate of SN per unit metal per hour.

d. Succinonitrile; **e.** Acetamide

REFERENCES

1. Zhou X, Qian K, Zhang Y *et al*. Tuning the size of photo-deposited metal nanoparticles via manipulating surface defect structures of TiO_2 nanocrystals. *Chemical Communications* 2020; **56**: 1964-1967.
2. Kresse G and Furthmüller J. Efficiency of ab-initio total energy calculations for metals and semiconductors using a plane-wave basis set. *Computational materials science* 1996; **6**: 15-50.
3. Blöchl P. Projector augmented-wave method. *Physical review B* 1994; **50**: 17953.
4. Perdew JP, Burke K, and Ernzerhof M. Generalized gradient approximation made simple. *Computational materials science* 1996; **77**: 3865.
5. Perdew J. Generalized gradient approximation made simple. *Phys. Rev. Lett* 1997; **77**: 3868.
6. Dudarev SL, Botton GA, Savrasov SY *et al*. Electron-energy-loss spectra and the structural stability of nickel oxide: An LSDA+ U study. *Physical Review B* 1998; **57**: 1505.
